# The striking incidence of animal listeriosis in Germany (2014–2024) indicates a persistent but neglected risk for One Health

**DOI:** 10.1186/s13567-025-01481-4

**Published:** 2025-03-08

**Authors:** Gamal Wareth, Heinrich Neubauer

**Affiliations:** Institute of Bacterial Infections and Zoonoses, Friedrich-Loeffler-Institut, Jena, Germany

**Keywords:** Listeriosis, animals, prevalence, distribution, Germany

## Abstract

Listeriosis is a serious zoonotic disease caused by the genus *Listeria*, with *Listeria monocytogenes* being the most pathogenic species for humans and various animal species. This bacterium is commonly found in the environment and poses significant health risks. We analysed official surveillance data detailing animal listeriosis in Germany over the last decade to unravel its host diversity and spatiotemporal distribution. Altogether, 1.629 notifications involving 3.326 various animal species were reported. Listeriosis has a broad host range in farm animals and wildlife, with a consistently striking incidence reported nationwide. Addressing this issue is crucial for public health and the safety of our food supply.

## Introduction, methods, and results

The genus *Listeria* (*L*.) currently encompasses 31 species and 8 subspecies, according to the List of Prokaryotic Names with Standing in Nomenclature (LPSN, accessed on January 20, 2025). Among these pathogens, only *L. monocytogenes* and *L. ivanoviii* are considered pathogenic for humans and animals [1]. *L. monocytogenes* is a Gram-positive, non-spore-forming, anaerobic, intracellular and zoonotic pathogen. It is capable of growing at low temperatures and producing biofilms. Therefore, it could develop into a particular risk for the food production industry through its ability to multiply in ready-to-eat products. It is a ubiquitous environmental pathogen that causes foodborne outbreaks in humans worldwide [2, 3]. Listeriosis is associated with high morbidity and mortality, especially in immunocompromised individuals with severe clinical manifestations, including gastroenteritis, encephalitis, meningitis, and septicaemia. Thus, listeriosis has become a disease with significant public health and economic consequences [4, 5]. Listeriosis in animals typically results in septicaemia, abortion, or latent infection [6, 7], and in ruminants, encephalitis is the most prominent form [7].

In Germany, several large outbreaks of human listeriosis have been reported in the last two decades [8–11], and almost all of these outbreaks are associated with the consumption of animal products. Thus, it has been extensively investigated along the food chain, starting at the slaughterhouse. However, in animals, it has received less attention than other bacterial foodborne pathogens, such as *Salmonella* or *Campylobacter.* However, for sustainable disease management, this notorious pathogen needs to be studied in the farm environment. Outbreaks in animals are an obvious starting point for this research. To address this gap, we analysed mandatory notification data concerning cases and outbreaks of animal listeriosis in Germany over 10 years (2014–2023). The spatiotemporal distribution and affected hosts across German federal states are described. Incomplete data from 2024 were also considered.

According to Germany’s Infection Protection Act (IfSG), *L. monocytogenes* in humans must be reported only when it is directly detected in blood, cerebrospinal fluid, other normally sterile substances, or swabs taken from newborns. Additionally, *L. monocytogenes* and *L. ivanovii* are classified as animal pathogens; however, listeriosis caused exclusively by *L. monocytogenes* is considered a notifiable animal disease in Germany. This means that all the data presented in the notification reports exclusively pertain to cases caused by *L. monocytogenes*.

**Figure 1:**
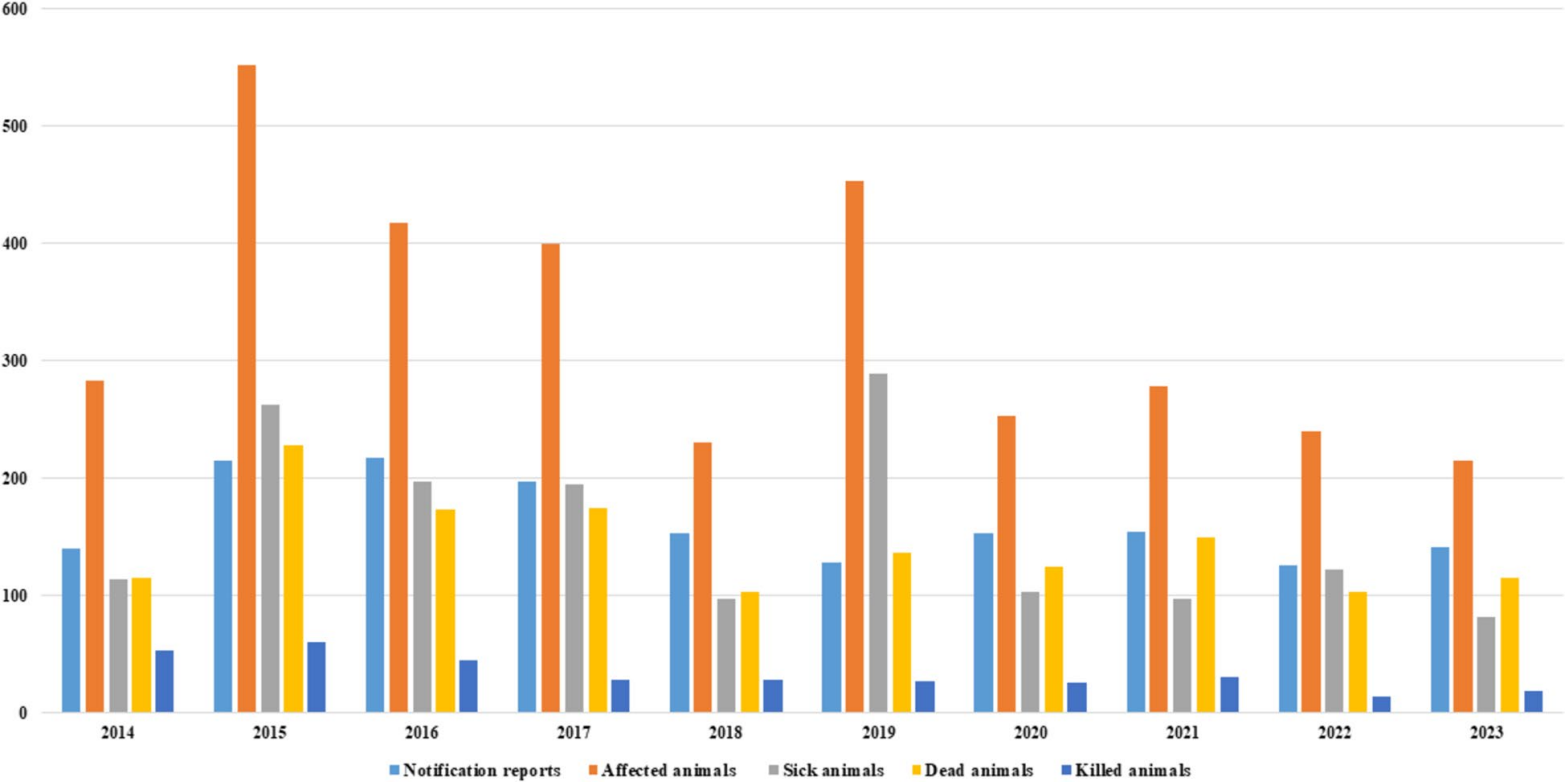
**Time trend distribution of listeriosis in affected animals in Germany (2014–2023), based on the number of notifications per year.**

**Figure 2:**
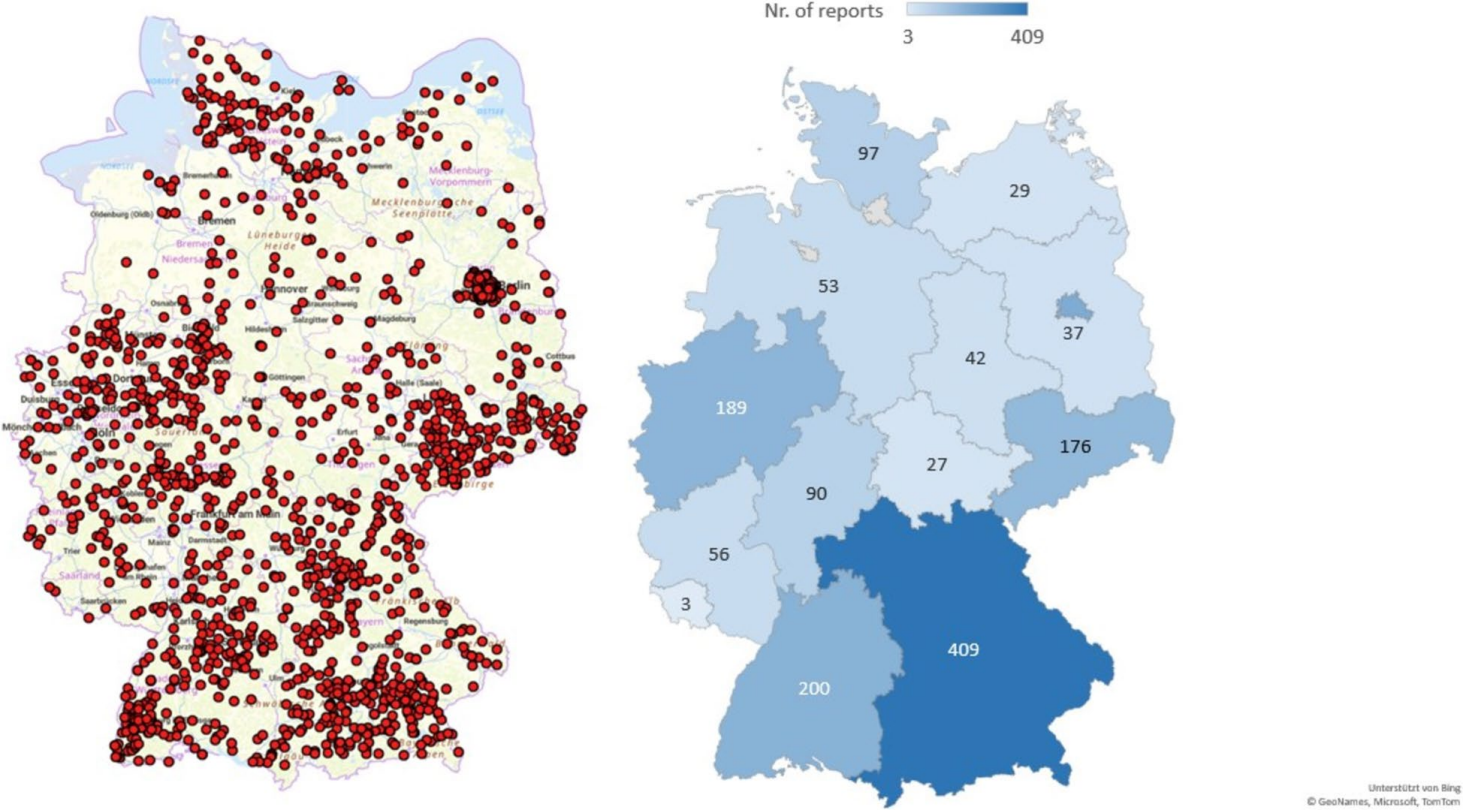
**Map of Germany showing the nationwide distribution of animal listeriosis in the last 10 years (2014–2023).**

The mandatory reporting data concerning cases and outbreaks of animal listeriosis over the last decade (2014–2023) have been analysed. The data were retrieved from the Animal Disease News System (TierSeuchenNachrichten-System, TSN). In total, 1629 notification reports (NRs) for listeriosis were registered, affecting 3326 animals, i.e., 1560 animals were sick, 1425 were dead, 333 were killed, and only 8 animals were slaughtered (Table 1). The highest annual incidence of affected animals was observed in 2015 (*n* = 552), followed by 2019 (*n* = 453), 2016 (*n* = 418), and 2017 (*n* = 400), with the lowest annual incidence occurring in 2023 (*n* = 216). The highest number of notifications was registered in 2016 (*n* = 218), and the lowest was registered in 2022 (*n* = 126). The temporal distribution of animal listeriosis is shown in Table 1 and Figure 1. The infections were distributed nationwide (Figure 2). Out of 16 federal states, 14 were affected and reported listeriosis in animals, whereas no cases were identified from the city states Hamburg and Bremen with marginal animal husbandry. Bavaria had the highest incidence of cases with 409 NRs, followed by Berlin (*n* = 221 NRs) and Baden-Wuerttemberg (*n* = 200 NRs). Saarland presented the lowest incidence of listeriosis with 3 NRs, followed by Thuringia (*n* = 27 NRs) and Mecklenburg-Pomerania (*n* = 29 NRs) (Table 1, Figure 2).

**Table 1:** **Spatiotemporal distribution of animal listeriosis in Germany (2014–2023), based on the annual number of notifications in each federal state**.

Year	2014	2015	2016	2017	2018	2019	2020	2021	2022	2023	Total
Notification reports	141	215	218	197	153	128	154	155	126	142	1.629
Affected animals	283	552	418	400	231	453	254	279	240	216	3.326
Sick animals	114	263	198	195	97	289	103	97	122	82	1.560
Dead animals	115	228	174	175	103	137	125	150	103	115	1.425
Killed animals	53	61	45	28	29	27	26	31	14	19	333
Slaughtered animals	1		1	2	2			1	1		8
Geographical distribution according to federal states											
Year	2014	2015	2016	2017	2018	2019	2020	2021	2022	2023	Total
Baden-Wuerttemberg	15	22	38	23	14	16	24	20	11	17	200
Bavaria	32	60	67	47	44	37	32	41	29	20	409
Berlin	8	10	8	40	21	12	31	30	26	35	221
Brandenburg	4	11	1	7	2	2	4	2	2	2	37
Hessia	8	17	9	9	3	11	8	7	10	8	90
Mecklenburg-Pomerania	1	4	6	4	5	5	2	1		1	29
Lower Saxony	9	10	9	5	6	4	4		4	2	53
North Rhine-Westphalia	13	25	23	16	14	16	15	28	18	21	189
Rhineland-Palatinate	5	8	2	9	9	3	3	5	7	5	56
Saarland	–	–	–	–	–	–	1	1	–	1	3
Saxony	18	23	31	22	17	11	14	13	11	16	176
Saxony-Anhalt	6	11	6	3	4	3	1	2	1	5	42
Schleswig–Holstein	20	8	14	9	12	5	15	3	4	7	97
Thuringia	2	6	4	3	2	3		2	3	2	27
Total	141	215	218	197	153	128	154	155	126	142	1.629

Listeriosis has been reported in almost all livestock but also in companion animals and wildlife. The highest number of cases was reported in cattle, followed by sheep and goats. Only 12 NRs were listed for pigs. Pet animal cases, i.e., dogs and cats, were reported in 25 and 8 NRs, respectively. Several wildlife species were mentioned, with foxes at the top with 179 NRs, followed by raccoons, fallow and roe deer, and rabbits. The names of the wild pigs are listed in the names of the 6 NRs. Poultry (chickens and pigeons) are also affected. Waterfowl (duck, 2016 and geese, 2023) have also been reported (Table 2).

Until the end of August 2024, 142 NRs encompassing 184 animals were received, equal to the number of notifications registered in 2023. Most of the cases were reported in sheep, cattle, foxes, and goats in sequence order. No cases have been reported in Brandenburg, but 32 NRs were registered for Berlin, 29 NRs for Bavaria, and 21 NRs for Baden-Wuerttemberg.

## Discussion

Numerous outbreaks of listeriosis have been reported in the human population globally [12–15], and human cases and outbreaks are frequently reported in Germany [8, 10, 11, 16–18]. Ingestion of contaminated food of animal origin is the main source of human infection. Listeriosis, or “circling disease”, is an acute disease that can arise in herds of small ruminants, particularly in winter when grass silage is fed. The disease is associated with significant economic losses due to abortion, loss of animals, and treatments. *L. monocytogenes* has a broad host range among animals nationwide in Germany, with more than 3300 affected animals in 10 years. Despite this fact, minimal attention has been given to outbreaks in animals, and little information is available on *L. monocytogenes* in animal environments [19–22]. The need for studies on the epidemiological situation in the veterinary sector is prominent. On the other hand, *L. ivanovii* poses a significant threat, as it can lead to severe infections and share virulence factors and pathogenic mechanisms with *L. monocytogenes*, underscoring the need for heightened awareness. This bacterium is also prevalent in nature and is found in the soil, water, plants, and faeces of various animals and birds. It can easily contaminate food products, including cheese, meat, fish, vegetables, and fruits, putting public health at risk. The genetic diversity of *L. ivanovii* and the variability in its virulence traits increase its pathogenic potential [23, 24]. Consequently, it is critical that we devote more resources to researching its epidemiology, distribution, detection, and virulence. Regular monitoring of its occurrence in animal farms, food, and the environment is essential, along with a deeper understanding of its molecular mechanisms of infection and adaptation to hosts. Moreover, emphasizing its inclusion in notification reports is vital to ensure swift public health responses.

**Table 2:** **Temporal distribution of listeriosis in different animal species in Germany over a period of 10 years (2014–2023)**.

Animal species	2014	2015	2016	2017	2018	2019	2020	2021	2022	2023	Total
Cattle	61	86	76	61	63	39	42	39	30	31	528
Sheep	35	51	67	43	38	43	31	39	30	39	416
Goats	13	25	26	17	20	17	20	12	20	17	187
Chickens	11	21	14	8	4	4	10	11	4	3	90
Dogs	–	–	3	1	2	3	1	7	3	5	25
Pigs/wild pigs	1	–/3	2	1/1	1	–/1	1/1	2	2	2	18
Equines	–	–	3	6	1	1	5	2	–	–	18
Rabbits	–	2	1	1	1	2	3	3	2	–	15
Cats	–	–	–	–	1	–	–	2	4	1	8
Alpaca	–	–	–	–	–	–	2	1	–	–	3
Time trend distribution of listeriosis in different wildlife species											
Fox	6	13	15	41	14	6	25	19	20	20	179
Racoon	2	–	1	7	7	4	8	10	7	16	62
Fallow deer	5	1	2	4		3	1	3	2		21
Roe deer	1	3	2	–	–	2	2	2	2	2	16
Hare	–	2	–	–	1	2	3	3	1	–	12

Dairy ruminants shed *Listeria* spp. in their faeces and may carry *L. ivanovii* in udders and *L. monocytogenes* in tonsils [1]. Shedding of the pathogen in faeces and subsequent colonization of tonsils and udders may explain the ubiquitous presence of *Listeria* in milk and a large variety of foods, as well as in soil, water, vegetation, animal feed, silage and sewage [20, 25]. *L. monocytogenes* was isolated from pig tonsil samples collected in abattoirs in Northwest and East Germany, highlighting the potential risk of contaminating pork meat [25]. The widespread of *L. monocytogenes* in livestock, companion animals, and wildlife [19–22] emphasizes the possible easy introduction of this notorious pathogen into the food chain and the potential risk of infection in humans. The susceptibility profiles of 259 *L. monocytogenes* strains collected over 40 years in Germany from patients, foods, and food-processing environments demonstrated that 38% were resistant to the last resort drug tigecycline and 56% (*n* = 145) were multidrug resistant (MDR) [26]. The drivers of resistance development are unknown and may include livestock, the food chain, humans, or the associated environment.

The impact of listeriosis on animal health and welfare still needs to be determined. Thus, systematic serogrouping, susceptibility testing, and whole-genome sequence typing of *Listeria* isolates from animals and their environments is necessary to help understand the epidemiological situation, detect drivers of resistance development, and ultimately protect humans. Eliminating sources of contamination will have the greatest effect on reducing the overall burden of listeriosis in Germany.

## Data Availability

Not applicable.
